# Current State of Adjuvant Hormone Therapy in Prostate Cancer and in the Management of Pelvic Nodal Recurrence: A Narrative Review

**DOI:** 10.3390/jcm15208047

**Published:** 2026-10-16

**Authors:** Barbara D’Andrea, Antonietta Montagna, Ilaria Benevento, Luciana Rago, Vito Metallo, Gerardo Morano, Melissa Romaniello, Fortunata Possidente, Vincenzo De Fina, Antonella Bianculli, Rosa Maria Carmela Lerose, Carmela Mazzoccoli, Donatella Telesca, Giovanni Storto, Rosj Gallicchio, Grazia Lazzari

**Affiliations:** 1 Radiation Oncology Unit, IRCCS CROB, 85028 Rionero in Vulture, PZ, Italy; barbara.dandrea@crob.it (B.D.); antonietta.montagna@crob.it (A.M.); ilaria.benevento@crob.it (I.B.); luciana.rago@crob.it (L.R.); vito.metallo@crob.it (V.M.); gerardo.morano@crob.it (G.M.); melissa.romaniello@crob.it (M.R.);; 2 Physic Unit, IRCCS CROB, 85028 Rionero in Vulture, PZ, Italy; vincenzo.defina@crob.it (V.D.F.); antonella.bianculli@crob.it (A.B.); 3 Pharmacy Unit, IRCCS CROB, 85028 Rionero in Vulture, PZ, Italy; rosa.lerose@crob.it (R.M.C.L.); carmela.mazzoccoli@crob.it (C.M.); donatella.telesca@crob.it (D.T.); 4 Nuclear Medicine Unit, IRCCS CROB, 85028 Rionero in Vulture, PZ, Italy; giovanni.storto@crob.it (G.S.); rosi.gallicchio@crob.it (R.G.)

**Keywords:** prostate cancer, androgen-deprivation therapy, hormone therapy, salvage radiotherapy, biochemical recurrence, metastasis-free survival

## Abstract

In post-prostatectomy prostate cancer, combining androgen deprivation therapy (ADT) with salvage radiotherapy (SRT) significantly improves clinical outcomes, though the absolute benefit varies based on baseline PSA, pathological risk, and treatment design. This review synthesizes findings from key randomized trials and contemporary meta-analyses—including RTOG 9601, SPPORT, GETUG-AFU 16, GETUG-AFU 22, and RADICALS-HD—evaluating the integration and duration of ADT with postoperative irradiation. While systemic therapy consistently enhances biochemical and clinical progression-free survival, a universal overall survival benefit remains uncertain or limited to high-risk cohorts with elevated pre-treatment PSA values. Concurrently, data from the prospective phase II OLIGOPELVIS (GETUG P07) and PEACE V-STORM trials are redefining the standard of care for pelvic nodal oligorecurrent disease detected via next-generation imaging (NGI). Transitioning away from exclusive lifelong ADT or isolated focal metastasis-directed therapy, emerging evidence strongly supports a multimodal paradigm: combining prostate bed RT, whole-pelvis elective nodal irradiation, and a high-dose boost to PET-positive nodes, complemented by a short course of systemic therapy. Ultimately, this narrative review provides clinicians with evidence-based guidance on integrating ADT to maximize regional disease control and delay systemic progression, while successfully sparing the majority of patients from the toxicities of continuous ADT.

## 1. Introduction

Androgen deprivation therapy (ADT) is a cornerstone of treatment for selected patients with intermediate- or high-risk prostate cancer receiving definitive or postoperative radiotherapy. In the definitive setting, the clinical utility of ADT is firmly established, as evidenced by the landmark MARCAP individual patient-data meta-analysis [1].

In contrast, the role of ADT after radical prostatectomy is more nuanced, and data remain limited regarding post-surgical pelvic nodal recurrence detected via next-generation imaging (NGI) [2]. Evidence from randomized trials and individual-patient meta-analyses indicates that the clinical impact of ADT is far from uniform. Its benefit is heavily influenced not only by the pre-treatment prostate-specific antigen (PSA) concentration at salvage but also by pathological grade and stage, surgical-margin status, the presence of nodal disease, radiation volume, and the specific duration and formulation of androgen suppression [3]. Intermediate endpoints—including biochemical progression-free survival, clinical progression-free survival, freedom from progression, and MFS (metastasis free survival)—consistently demonstrate greater improvement than OS as shown in POSEIDON individual patient-data meta-analysis [4].

In this individual patient-data meta-analysis, the addition and duration of hormone therapy combined with postoperative radiotherapy (PORT) did not yield a statistically significant overall survival benefit. However, this effect was strongly modulated by pre-radiotherapy PSA levels: no meaningful OS benefit was observed at PSA values ≤ 0.5 ng/mL, whereas a potential survival advantage from long-term therapy emerged only at substantially higher PSA thresholds [4].

With respect to early pelvic nodal recurrence, locoregional salvage treatment combined with ADT has become highly feasible due to the diagnostic accuracy of modern imaging modalities. The introduction of NGI, particularly prostate-specific membrane antigen positron-emission tomography (PSMA-PET), has driven a major clinical paradigm shift. By identifying pelvic nodal or distant recurrences at significantly lower PSA levels than conventional imaging, NGI creates vital opportunities for early, tailored salvage interventions. However, it also introduces challenges, notably the risk of stage migration and artificial shifts in patient prognosis [5,6]. Consequently, this narrative review discusses the current evidence for combining ADT with postoperative radiotherapy and examines contemporary management strategies for pelvic nodal oligorecurrence.

## 2. Methods

This narrative review was structured to synthesize clinically relevant evidence rather than to perform a formal systematic review or quantitative meta-analysis. A comprehensive literature search was conducted across the PubMed/MEDLINE, Embase, and Cochrane Library databases for articles published in the last eight years up to 2026 using combinations of the following medical subject headings (MeSH) and text words: “prostate cancer,” “androgen deprivation therapy,” “hormone therapy,” “salvage radiotherapy,” “postoperative radiotherapy,” “metastasis-free survival,” “oligorecurrence,” “elective nodal irradiation,” and “next-generation imaging.”

Eligibility criteria encompassed randomized controlled trials, prospective cohorts, retrospective studies, systematic reviews, and meta-analyses evaluating the integration of ADT with definitive, adjuvant, or salvage radiotherapy. Particular emphasis was placed on trials reporting overall survival (OS), metastasis-free survival (MFS), distant metastasis, biochemical or clinical progression, toxicity, and quality-of-life outcomes. Studies investigating pelvic nodal oligorecurrence detected via NGI were also prioritized. Conversely, preclinical studies, case reports, duplicate publications, and manuscripts lacking detailed descriptions of radiotherapy or ADT schedules were excluded from this analysis. Given the narrative nature of this synthesis, formal risk-of-bias assessment and pooled statistical analyses were not performed. No artificial intelligence (AI) tools were utilized in the drafting or literature curation of this manuscript.

## 3. Clinical Evidence for Hormone Therapy with Postoperative Radiotherapy

Study endpoints varied across the included literature, encompassing event-free survival (EFS), progression-free survival (PFS), metastasis-free survival (MFS), distant metastasis (DM), freedom from progression (FFP), and cancer-specific mortality (CSM). Crucially, these endpoints—particularly MFS, biochemical control, and clinical progression—should not be interpreted as interchangeable clinical outcomes. Furthermore, baseline PSA levels, radiation doses, and target volumes demonstrated substantial heterogeneity among the analyzed trials. Detailed characteristics and outcomes of these studies are summarized in Table 1.

### 3.1. Clinically Relevant Endpoints

Postoperative radiotherapy is utilized either as adjuvant treatment for adverse pathological findings or as early salvage treatment for a rising or persistent PSA level. The patient populations enrolled in the major clinical trials differed substantially regarding baseline PSA thresholds, pathological characteristics, the use of pelvic lymph-node dissection, imaging modalities, radiation fields, and systemic regimens. These confounding differences inherently limit direct comparisons between studies. In modern trial design, metastasis-free survival (MFS) is increasingly used as a clinically meaningful endpoint and has been proposed as a surrogate for overall survival (OS) across most prostate cancer research [7]. Historically, OS served as the primary endpoint solely in the RTOG 9601 trial, in which a secondary analysis formally evaluated MFS as a surrogate for OS [8]. Overall, the POSEIDON meta-analysis analyzed individual data from more than 6000 patients across various primary outcomes including event-free survival (EFS), progression-free survival (PFS), MFS, distant metastasis (DM), freedom from progression (FFP), and cancer-specific mortality over a median follow-up of 12 years [4].

### 3.2. Patient Selection

Patient characteristics were highly heterogeneous across the analyzed studies with respect to surgical approach, baseline prostate-specific antigen (PSA) levels, and radiation doses and volumes. Patients staged via conventional imaging were generally eligible if they had undergone radical prostatectomy, with or without lymphadenectomy, and presented with either a persistent postoperative PSA or biochemical recurrence (defined as two consecutive PSA values ≥ 0.2 ng/mL). Additional inclusion criteria encompassed high-risk features such as pathological T3b/T4 stage, positive surgical margins (R1), a Gleason score ≥ 8, and a pre-androgen deprivation therapy (ADT) PSA value ranging from a minimum value of 0.1–0.2 ng/mL up to 2–4 ng/mL, depending on the protocol. Pelvic lymphadenectomy was mandatory solely in the RTOG 9601 trial; in the SPPORT trial, 65% of patients underwent nodal dissection with a median of 6 lymph nodes removed (range, 3–11), whereas this staging procedure was not specified in the RADICALS-HD trial [7,8]. Furthermore, entry PSA levels lacked uniformity across trials, with median cohort values spanning a broad spectrum from ≥0.5 ng/mL to ≥1.6 ng/mL.

The baseline PSA level at the time of salvage radiotherapy represents a critical clinical variable for treatment selection. In the multi-trial analyses summarized by Spratt et al. (the POSEIDON meta-analysis), the overall survival (OS) benefit associated with the addition of ADT was predominantly concentrated in patients with a pre-treatment PSA > 0.5 ng/mL (*p* = 0.02). Among patients randomized to the postoperative radiotherapy (PORT) arm receiving long-term ADT, a significant OS benefit was observed specifically in cases with a baseline PSA > 1.6 ng/mL, whereas the survival advantage was negligible at lower PSA thresholds [4].

Subgroup analyses from the RTOG 9601 trial similarly demonstrated that long-term antiandrogen therapy yielded the largest absolute clinical benefit in patients with entry PSA values above approximately 1.5 ng/mL [8]. Specifically, in the RTOG 9601 cohort with a baseline PSA > 1.5 ng/mL, the 18-year metastatic disease rate was significantly lower at 17.4% in the hormone therapy arm compared to 40.6% in the placebo group (*p* = 0.004). Conversely, in the intermediate subgroup with a PSA between 0.7 and 1.5 ng/mL, the metastatic rate was 23.8% versus 31.7%, failing to reach statistical significance (*p* = 0.07). In the SPPORT trial, where the pre-ADT PSA cutoff was set at 0.35 ng/mL (serving as the median reference value for freedom from progression (FFP)), patients with a PSA >0.35 ng/mL achieved the most favorable FFP outcomes in Arm 3 (pelvic radiotherapy plus ADT) compared to Arm 2 (prostate bed radiotherapy plus ADT), though this specific difference did not cross the threshold for statistical significance [9].

### 3.3. Which Radiotherapy Modality?

The radiotherapy protocols evaluated in this review consisted of high-technology external beam radiotherapy (EBRT) delivered with salvage intent to all patients. Regarding treatment volumes, the prostate bed represented the primary target volume across most trials, with or without the inclusion of regional pelvic lymph nodes. Elective pelvic nodal irradiation was routinely incorporated in both GETUG trials [10,11] and, most notably, in Arm 3 of the SPPORT trial, whereas its use was left to institutional discretion in the RADICALS-HD study [12]. Concerning dose-fractionation schedules, a conventional fractionation regimen delivering a total dose ranging from 64.8 to 70.2 Gy was adopted across the majority of studies. A notable exception was the RADICALS trial, which permitted a moderate hypofractionation schedule of 52.5 Gy in 20 fractions as an alternative to a conventionally fractionated regimen of 66 Gy in 30 fractions [13]. When pelvic lymph nodes were included in the target volume, the prescribed elective nodal dose was 45 Gy.

## 4. Adjuvant ADT Across the Studies

The included trials varied significantly in their comparative designs, evaluating either radiotherapy alone versus radiotherapy combined with ADT or short-term versus long-term ADT schedules. The systemic regimens also differed across studies, utilizing various hormonal agents including bicalutamide, flutamide, goserelin, triptorelin, or degarelix. Five pivotal randomized controlled trials—RTOG 9601, SPPORT, GETUG-AFU 16, GETUG-AFU 22, and RADICALS-HD have fundamentally established the role of combining SRT with ADT in recurrent prostate cancer, demonstrating that the addition of systemic therapy consistently improves metastasis-free survival (MFS), particularly in the presence of adverse risk factors [8,9,10,11,12]. While the SPPORT trial highlights a distinct clinical benefit from expanding the treatment volume to include elective pelvic nodal irradiation, trials like RADICALS-HD help define the optimal duration of hormone therapy and underscore the critical need for risk-adapted patient selection to balance oncological control against treatment-related toxicities [8,12]. When comparing RTOG 9601 with the long-term assessment of the RADICALS-HD trial (Arm 2 vs. Arm 3), several key similarities emerge despite distinct chronological and design differences. First, both protocols evaluated the therapeutic impact of combining ADT with SRT in patients presenting with recurrent or persistent PSA levels following radical prostatectomy. Second, both studies aimed to define the optimal duration of systemic treatment to maximize therapeutic intensity without compromising safety. Moreover, both trials integrated mature, long-term follow-up to rigorously assess definitive survival endpoints, specifically overall survival (OS) and MFS within the same post-surgical clinical setting [8,12].

### 4.1. Short-Course Hormone Therapy Versus Radiotherapy Alone

The GETUG-AFU 16 trial evaluated the efficacy of salvage radiotherapy (SRT) with or without a short course of goserelin. The study demonstrated a significant progression-free survival (PFS) benefit across both low- and high-risk disease categories. At 10 years, the overall PFS rates were 64% in the goserelin combination arm versus 49% with radiotherapy alone (*p* < 0.0001). Within the low-risk stratum, the 10-year PFS was 74% in the combination arm compared to 61% in the radiotherapy-alone arm (*p* = 0.0041); in the high-risk stratum, the rates were 60% versus 43%, respectively (*p* < 0.0001). Furthermore, the 10-year metastasis-free survival (MFS) significantly favored the combined modality arm (75% vs. 69%; *p* = 0.0339), although no significant interaction was observed between risk categories. Conversely, overall survival (OS) at 120 months remained highly comparable between the radiotherapy plus goserelin and radiotherapy-alone groups (86% vs. 85%; *p* = 0.73). The incidence of adverse events was similar between the two cohorts; notably, long-term urinary incontinence occurred in 4% of patients receiving combination therapy versus 5% of those treated with radiotherapy alone [10].

The GETUG-AFU 22 trial was an investigator-initiated, randomized, multicenter, phase II study designed to evaluate the efficacy and safety of adding short-term ADT, specifically utilizing the GnRH antagonist degarelix, to salvage radiotherapy (SRT). The study reported no statistically significant difference in 5-year event-free survival (EFS) between the radiotherapy-alone and radiotherapy plus ADT arms (62.3% vs. 63.5%, respectively; *p* = 0.528). Similarly, a minimal, non-significant variance was noted in biochemical progression-free survival (bPFS), which reached 62.3% with radiotherapy alone versus 66.0% with the combined regimen (*p* = 0.322). In contrast, metastasis-free survival (MFS) was significantly improved in the combination arm (*p* = 0.048). Regarding safety, while acute toxicity profiles were highly comparable, a significantly higher rate of overall toxicity at 6 months post-radiotherapy was observed in the combination group (70.5% vs. 53.1%; *p* = 0.046). However, long-term health-related quality of life, assessed at 24 months via the EORTC QLQ-C30 and QLQ-PR25 questionnaires, showed no significant differences between the two treatment cohorts [11].

The SPPORT trial evaluated three distinct treatment strategies: prostate-bed radiotherapy (PBRT) alone (Arm 1), PBRT plus short-term ADT (Arm 2), and PBRT combined with pelvic nodal radiotherapy and short-term ADT (Arm 3). Regarding the protocol-specified 4–6 months of ADT, investigators were permitted to utilize standard, guideline-recommended luteinizing hormone-releasing hormone (LHRH) agonists, predominantly leuprolide or goserelin. The primary endpoint was 5-year freedom from progression (FFP), defined as biochemical failure according to the Phoenix criteria (nadir + 2 ng/mL). The 5-year FFP rates were 71.1% in Arm 1, 81.3% in Arm 2, and 87.4% in Arm 3. In the pairwise comparisons, the adjusted hazard ratio (adj-HR) was 0.50 for Arm 3 versus Arm 1, 0.60 for Arm 2 versus Arm 1, and 0.82 for Arm 3 versus Arm 2. Notably, the 5-year FFP in Arm 3 was statistically superior to both Arm 1 and Arm 2, demonstrating an absolute increment of 6.1% over Arm 2 (*p* = 0.003). Regarding distant metastasis (DM), the HR was 0.55 for Arm 3 versus Arm 1 (*p* < 0.001), 0.78 for Arm 2 versus Arm 1 (*p* = 0.083), and 0.70 for Arm 3 versus Arm 2 (*p* = 0.043). For overall survival (OS), the adj-HR was 0.93 for Arm 3 versus Arm 1, 0.87 for Arm 2 versus Arm 1, and 1.07 for Arm 3 versus Arm 2. Consequently, Arm 3 yielded superior clinical outcomes across multiple endpoints, albeit at the expense of a significantly higher rate of acute grade 2+ and 3+ toxicities compared to Arms 1 and 2 [9].

In the RADICALS-HD trial, the protocol mandated the use of a luteinizing hormone-releasing hormone (LHRH) agonist at the investigator’s discretion, such as triptorelin, leuprolide, or goserelin. Exceptionally, and exclusively outside of Canada, oral bicalutamide at a dose of 150 mg/day or degarelix was accepted as an alternative regimen. The short-course comparison evaluated radiotherapy alone (Arm 1: no ADT) versus radiotherapy plus 6 months of ADT (Arm 2: short ADT) to determine whether adding short-term systemic therapy improves oncological outcomes. The primary endpoint for this comparison was 10-year metastasis-free survival (MFS). Over a median follow-up of 9.0 years, adding 6 months of ADT did not significantly improve MFS compared to radiotherapy alone (HR 0.886, 95% CI 0.699–1.123; *p* = 0.35), with 10-year MFS rates reaching 80.4% in the short-course ADT arm versus 79.2% in the no-ADT group. However, clinical progression-free survival (cPFS) was significantly improved in the combination arm, demonstrating a hazard ratio of 0.544 (95% CI 0.433–0.684; log-rank *p* < 0.0001). Conversely, no statistically significant differences were detected regarding overall survival (OS), freedom from distant metastasis (FFDM), or severe treatment-related toxicities, with RTOG grade 3 or higher adverse events reported in 14% of the short-course ADT group versus 17% in the radiotherapy-alone group (*p* = 0.15) [12].

### 4.2. Long-Course ADT Versus Placebo

The landmark RTOG 9601 trial established the efficacy of long-term antiandrogen monotherapy using oral bicalutamide at a dose of 150 mg daily for 2 years versus placebo combined with salvage radiotherapy (SRT). In the mature 18-year follow-up analysis, the addition of hormone therapy yielded a significant overall survival (OS) benefit, with survival rates reaching 53% in the combination arm versus 43% in the placebo group (*p* = 0.025). Furthermore, a significantly lower incidence of long-term metastatic disease was observed at 22% in the ADT arm compared to 31% with placebo (*p* = 0.001).

Subgroup analyses revealed that the therapeutic benefit was strongly modulated by baseline risk factors. Among patients with elevated pre-treatment PSA levels (1.5–4.0 ng/mL), the 18-year metastatic disease rate was dramatically reduced to 17.4% in the combined group versus 40.6% in the placebo group (*p* = 0.004). Similarly, in patients with a Gleason score (GS) of 8–10, the metastatic rate was 24.3% in the combination arm versus 53.6% in the placebo arm (*p* < 0.0001). For patients presenting with positive surgical margins (R1), the incidence of distant metastasis was 17.8% in the combination arm versus 27.2% with placebo (*p* = 0.001). Conversely, in the intermediate subgroup with entry PSA values between 0.7 and 1.5 ng/mL, the rate was 23.8% versus 31.7%, failing to reach statistical significance (*p* = 0.07). Regarding safety, bicalutamide was characteristically associated with an increased incidence of breast-related adverse effects, specifically gynecomastia and breast pain [8].

### 4.3. Short Course ADT Versus Long Course ADT

In the second main comparison of the RADICALS-HD trial, patients were randomized to receive either short-course ADT (Arm 2; 6 months) or long-course ADT (Arm 3; 24 months), with the latter consisting of an extended 2-year course of hormone therapy. Over a mature follow-up, long-course ADT significantly improved 10-year metastasis-free survival (MFS) and delayed the need for subsequent systemic treatments. However, this absolute survival benefit was modest and must be carefully weighed against the cumulative metabolic, cardiovascular, sexual, skeletal, and quality-of-life toxicities inherently associated with prolonged androgen suppression.

In the direct comparison between long-term and short-term ADT, the 10-year MFS rates were 78.1% versus 72.1%, respectively, demonstrating a statistically significant advantage favoring the 24-month regimen (HR 0.773, 95% CI 0.612–0.975; log-rank *p* = 0.029). Long-term ADT also significantly improved freedom from distant metastasis (FFDM) (HR 0.68, 95% CI 0.53–0.88; *p* = 0.0024) and cPFS (HR 0.68, 95% CI 0.53–0.87; *p* = 0.0024). Interestingly, the relative advantage in terms of preventing distant-metastasis events was less pronounced than that observed in the ‘zero-to-six months’ comparison. Furthermore, a significantly delayed time to subsequent non-protocol salvage ADT emerged in the long-term ADT arm (HR 0.73, 95% CI 0.59–0.91), confirming more durable regional and systemic disease control [12].

## 5. Hormone Therapy and Pelvic Nodal Recurrence

The management of oligometastatic pelvic nodal recurrence, typically defined as the presence of a maximum of 3 to 5 positive lymph nodes and currently almost exclusively detected via Next-Generation Imaging (NGI) such as PSMA-PET, represents an area of major clinical transition. This scenario differs from conventional imaging-detected biochemical recurrence because the clinician can identify macroscopic nodal disease and may tailor both the radiation volume and the systemic regimen.

Historically, these patients were managed with long-term androgen deprivation therapy (ADT) alone. Today, the integration of targeted radiotherapy strategies like Metastasis-Directed Therapy (MDT) [14,15,16] and Elective Nodal Irradiation (ENI), with or without short-course ADT, is supported by several pivotal prospective randomized clinical trials.

The rationale for ENI is that visible nodal lesions may represent part of a wider field of microscopic regional disease. ENI, combined with a simultaneous integrated boost (SIB) to the involved nodes detected by NGI, may therefore provide a more comprehensive regional treatment compared to stereotactic body radiotherapy (SBRT) directed solely at visible lesions. Tumor bed radiotherapy is also permitted if not previously irradiated. Furthermore, short-course ADT can provide systemic cytoreduction and may enhance the efficacy of radiotherapy. Within this setting, two phase II trials are assessing the efficacy of this triplet combination: the OLIGOPELVIS (GETUG P07) prospective trial and the PEACE V-STORM randomized trial [17,18].

### 5.1. OLIGOPELVIS Study (GETUG P07): Prospective Trial

The OLIGOPELVIS study (GETUG P07) evaluated patients with pelvic nodal recurrence presenting with up to 5 positive lymph nodes on Choline-PET. Treatment consisted of high-dose intensity-modulated radiotherapy (IMRT) delivering 54 Gy in 30 fractions to the pelvis and a simultaneous integrated boost of 66 Gy in 30 fractions to the positive lymph nodes, combined with 6 months of long-acting ADT. The primary endpoint was the 2-year progression-free survival (PFS) rate, defined as freedom from biochemical recurrence (two consecutive prostate-specific antigen levels above the baseline at inclusion), radiological or clinical progression (per RECIST 1.1), or death from any cause.

After a median follow-up of 6.1 years in a cohort of 67 patients, the trial reported the following outcomes: at 2 years, the PFS rate was 81%, and the biochemical relapse-free survival (BRFS) rate was 58%. The median PFS was 45.3 months. At 5 years, the PFS rate was 39%, the BRFS rate was 31%, and the overall survival (OS) rate reached 90.9%. The 5-year ADT-free survival ranged between 58% and 64%, indicating that the majority of patients successfully avoided continuous systemic treatment. Late grade 2+ toxicities remained low, with genitourinary (GU) and gastrointestinal (GI) event rates of 4% to 12% and 3% to 4%, respectively [17].

### 5.2. PEACE V-STORM: Randomized Trial

In the PEACE V-STORM randomized trial, patients presenting with pelvic nodal oligorecurrent prostate cancer (defined as ≤5 positive lymph nodes confined to the pelvis) detected exclusively via next-generation imaging (NGI), specifically choline or PSMA-PET/CT following definitive local treatment, were enrolled. Out of 196 randomized patients, 190 were evaluable and included in the final analysis. Based on the principle that metastasis-free survival (MFS) serves as a valid surrogate for overall survival (OS), the primary endpoint was MFS, defined as the time from randomization to the detection of distant metastases (outside the pelvis) on imaging or death from any cause.

Concomitant systemic therapy was identical in both arms, consisting of a mandatory 6-month short course of ADT. The radiotherapy strategies differed as follows: Arm A (Focal Therapy/Control Group): Metastasis-Directed Therapy (MDT) alone, consisting of either stereotactic body radiotherapy (SBRT/SABR) or salvage lymph node dissection (sLND) targeting only the PET-positive nodes; Arm B (Multimodal Therapy/Experimental Group): Elective Nodal Radiotherapy (ENRT) to the whole pelvis (45 Gy) combined with a simultaneous integrated boost (SIB) to the macroscopic PET-positive lymph nodes (dose-escalated up to 65 Gy).

The trial demonstrated a numerically higher MFS at 4 years with pelvic ENRT plus SIB compared to MDT alone (76% versus 63%, respectively; *p* = 0.063). Because of this *p*-value, the difference represents a favorable trend rather than a statistically significant result. Consequently, longer follow-up and confirmatory studies are required before this strategy can be considered definitive for all patients with pelvic nodal oligorecurrence. At long-term follow-up, cumulative late grade 2 toxicities were low and comparable between both treatment groups, with no reported grade 4 or 5 adverse events. Health-related quality of life (HRQoL) scores dipped transiently during treatment due to acute radiation and short-term ADT side effects but fully recovered to baseline levels across both arms within 12 to 24 months [18]. Table 2 outlines the main characteristics and differences.

## 6. Discussion

Current evidence supports a risk-adapted approach to androgen deprivation therapy (ADT) combined with postoperative salvage radiotherapy (SRT). The POSEIDON analysis demonstrated that the principal benefit of adjuvant ADT is most consistently observed in biochemical, clinical, and metastasis-related endpoints, rather than translating into a universal overall survival (OS) advantage [4]. Although patients enrolled in these studies underwent postoperative SRT according to the ARTISTIC meta-analysis [19], several biases must be highlighted because a “one-size-fits-all” analysis confounds the interpretation of these data as follows.

Heterogeneous systemic agents were delivered: the included trials utilized treatments ranging from oral bicalutamide and luteinizing hormone-releasing hormone (LH-RH) agonists to oral LH-RH antagonists, thereby introducing substantial confounding factors regarding toxicity profiles.

Baseline prostate-specific antigen (PSA) eligibility thresholds varied significantly across protocols: for instance, certain trials assumed a benefit threshold at 0.5 ng/mL, whereas the SPPORT trial established a pre-hormone therapy (HT) PSA cutoff of 0.35 ng/mL [9].

The impact of early intervention is not clear because available analyses suggest that early SRT initiated at low PSA levels may allow selected patients to forgo prolonged HT [20]. Conversely, patients presenting with higher PSA levels, high-grade disease, seminal vesicle invasion, extraprostatic extension, positive surgical margins, or other adverse features derive greater benefit from systemic intensification [12]. Nonetheless, the absolute PSA value to consider ADT intervention is insufficient. As defined by the 2025 European Association of Urology (EAU) guidelines, risk stratification must incorporate additional parameters, including a PSA doubling time (PSADT) < 1 year, ISUP grade group 4–5, and a time to biochemical recurrence (BCR) < 18 months [3].

Moreover, there are methodological disparities when considering that pelvic lymphadenectomy was not uniformly performed, and radiation fields, total doses, and fractionation schedules varied across studies. Importantly, there are statistical and technological gaps. In fact, these trials were not powered for identical endpoints, and all were conducted prior to the widespread adoption of prostate-specific membrane antigen positron emission tomography (PSMA-PET). Current guidelines now recommend PSMA-PET imaging during post-surgical BCR, even at very low or persistently detectable PSA values [3].

Despite these limitations, actionable data exist regarding the optimization of ADT duration, which must be strictly individualized as follows. A 6-month course could be appropriate for selected patients with intermediate risk where the primary objectives are radiosensitization and short-term systemic control. A 24-month course may be considered for patients at high risk of distant failure, particularly those with higher PSA levels and unfavorable pathological features. Definitive data regarding an intermediate 18-month duration are eagerly awaited. However, clinical decisions must integrate patient age, cardiovascular and metabolic risk profiles, bone health, sexual function, cognitive and emotional well-being, and patient preferences.

Finally, the introduction of next-generation imaging (NGI) to identify nodal recurrence is critical. It detects high-risk patients who remain candidates for local salvage strategies without early concurrent androgen receptor pathway inhibitor (ARSI) combination, as conducted in the EMBARK trial [21]. In NGI-defined nodal recurrence, the choice between focal metastasis-directed therapy (MDT) and elective nodal irradiation (ENI) remains an area of active investigation rather than an established standard of care. Nevertheless, encouraging data support combining ENI with MDT and short-term HT. Mature results from the OLIGOPELVIS study, featuring a median follow-up exceeding 6 years, now complement the comparative, though less mature, findings reported by the STORM investigators [17,18].

## 7. Future Perspectives

Future investigations on this topic should utilize next-generation imaging (NGI)-guided patient selection and treatment customization. Tools like PSMA-PET and other highly sensitive imaging modalities significantly improve recurrence localization, supporting the individualized selection of prostate-bed radiotherapy, elective nodal irradiation (ENI), nodal boosts, or metastasis-directed therapy (MDT). To generate robust comparative evidence, future trials must achieve greater homogeneity in patient selection, particularly regarding surgical approaches and radiotherapy optimization. Moreover, in the era of biomarker-driven personalization, the integration of novel prognostic tools is essential, such as advanced biomarkers and Patient-Reported Outcomes (PROs).

Advanced Biomarkers like genomic classifiers, circulating tumor DNA (ctDNA), and circulating tumor cells (CTCs) hold the potential to accurately distinguish patients who require treatment intensification from those who can safely undergo treatment de-escalation; however, rigorous prospective validation remains essential [22,23]. Alongside traditional oncological endpoints, future studies must systematically incorporate PROs to evaluate impact on quality of life, sexual function, fatigue, as well as metabolic, cardiovascular, skeletal, and cognitive health [24].

## 8. Conclusions

Androgen deprivation therapy (ADT) combined with postoperative salvage radiotherapy (SRT) remains an effective strategy for selected patients experiencing biochemical recurrence (BCR) after radical prostatectomy. Its clinical benefits are most consistently observed in progression- and metastasis-related outcomes, whereas overall survival (OS) advantages vary depending on baseline risk stratification and prostate-specific antigen (PSA) levels. To guide clinical decision-making, current evidence indicates that short-course ADT, when combined with pelvic nodal irradiation, improves disease control in appropriately selected patients, though it increases acute toxicity profiles, while long-course ADT may improve metastasis-free survival (MFS) in patients at higher risk of distant failure. However, it carries clinically relevant long-term morbidity. For regional recurrence detected by next-generation imaging (NGI), elective nodal irradiation (ENI) combined with a boost to the involved nodes and short-course ADT represents a biologically plausible and promising approach. Nonetheless, this evidence should be interpreted cautiously until mature confirmatory data from phase III trials are available. Consequently, treatment must be strictly individualized, integrating PSA kinetics (e.g., doubling time), pathological features, advanced imaging findings, anticipated toxicity, patient comorbidities, and personal preferences.

## Figures and Tables

**Table 1:** Overview of randomized trials evaluating hormone therapy with postoperative salvage radiotherapy.

Trial	Population/Design	HT Regimen	Radiotherapy	Principal Reported Outcomes	Key Toxicity/QoL Considerations
GETUG-AFU 16	Phase III RCT; BCR after prostatectomy	Goserelin for 6 months vs. placebo	Prostate bed; 66 Gy in the source data	10-y PFS: 64% vs. 49%; 10-y MFS: 75% vs. 69%; OS not significantly different	Late urinary toxicity broadly comparable in the summarized data
GETUG-AFU 22	Randomized trial; BCR/high-risk features	Short-course antiandrogen for 6 months vs. placebo	Prostate bed ± pelvic nodes	5-y EFS: 63.5% vs. 62.3%; MFS favored HT in the reported analysis	More toxicity at 6 months with HT; no persistent QoL difference reported at 24 months
SPPORT	Phase III multi-arm RCT; post-prostatectomy BCR	Short-course HT in Arms 2 and 3	Arm 1: bed; Arm 2: bed + HT; Arm 3: bed + pelvic nodes (45 Gy) + HT	5-y FFP: 71%, 81%, and 87%; distant metastasis reduced in Arm 3 vs. Arm 1	Larger fields increased acute grade ≥ 2/3 GU and GI toxicity
RTOG 9601	Phase III RCT; salvage radiotherapy after prostatectomy	Bicalutamide 150 mg daily for 24 months vs. placebo	Prostate bed; 64.8–68.4 Gy in the source data	Long-term OS and distant-metastasis benefit, greatest in higher-PSA and adverse-risk subgroups	Gynecomastia and breast tenderness increased with bicalutamide
RADICALS-HD	Factorial randomized trial after prostatectomy	No HT vs. 6 months; 6 vs. 24 months	Conventional or moderate hypofractionation	No-HT vs. 6-month comparison: no significant 10-y MFS difference; 24 vs. 6 months: improved 10-y MFS with longer HT	No significant difference in reported grade ≥ 3 late toxicity in the summarized comparison

Abbreviations: BCR, biochemical recurrence; EFS, event-free survival; FFP, freedom from progression; GI, gastrointestinal; GU, genitourinary; HT, hormone therapy; MFS, metastasis-free survival; OS, overall survival; PFS, progression-free survival; RCT, randomized controlled trial.

**Table 2:** Pelvic nodal oligorecurrence and treatment-selection considerations.

Feature	PEACE V-STORM Trial	OLIGOPELVIS Trial (GETUG P07)
**Study Design**	International, randomized, phase II trial	Prospective, single-arm, multicenter phase II trial
**Clinical Question**	Focal MDT (SBRT/sLND) vs. Extended Nodal RT (ENRT)	Efficacy of early multimodal treatment (ENRT + ADT)
**Randomization**	Yes (1:1 ratio)	No (all patients received the same protocol)
**Sample Size**	196 randomized (190 analyzed)	74 enrolled (67 analyzed)
**Oligometastasis Criteria**	≤5 pelvic lymph nodes	≤5 pelvic lymph nodes
**Imaging Modality (NGI)**	Choline-PET/CT or PSMA-PET/CT	18F-Choline-PET/CT exclusively
**Control Arm**	Arm A: Focal MDT (SBRT or sLND) to PET-positive nodes only	None (single-arm design)
**Experimental Arm**	Arm B: Whole-pelvis ENRT (45 Gy) + SIB to PET-positive nodes (up to 65 Gy)	Whole-pelvis ENRT (45 Gy) + High-dose boost to PET-positive nodes (66–72 Gy)
**Systemic Therapy (ADT)**	Mandatory 6 months of ADT (both arms)	Mandatory 6 months of ADT (all patients)
**Primary Endpoint**	Metastasis-Free Survival (MFS) at 4 years	Progression-Free Survival (PFS) at 2 years
**Data Maturity**	4-year follow-up results	6.1-year median follow-up (highly mature)
**Key Efficacy Outcomes**	4-year MFS: 76% (Arm B) vs. 63% (Arm A) (*p* = 0.063)	2-year PFS: 81% 5-year PFS: 39% 5-year OS: 90.9%
**ADT-Free Survival**	Evaluated as a secondary endpoint	5-year ADT-free survival: 58–64%
**Toxicity Profile**	Higher acute Grade 2+ GI/GU in Arm B; low and equivalent late toxicities	Acute Grade 2+: 10% GU, 2% GI Late Grade 2+: 4–12% GU, 3–4% GI

## Data Availability

Data are available in the References section.
